# Training intensity quantification of core stability exercises based on a smartphone accelerometer

**DOI:** 10.1371/journal.pone.0208262

**Published:** 2018-12-05

**Authors:** David Barbado, Belen Irles-Vidal, Amaya Prat-Luri, María Pilar García-Vaquero, Francisco J. Vera-Garcia

**Affiliations:** Sports Research Centre, Miguel Hernandez University of Elche, Elche, Alicante, Spain; Sao Paulo State University - UNESP, BRAZIL

## Abstract

Although core stability (CS) training is largely used to enhance motor performance and prevent musculoskeletal injuries, the lack of methods to quantify CS training intensity hinders the design of CS programs and the comparison and generalization of their effects. The aim of this study was to analyze the reliability of accelerometers integrated into smartphones to quantify the intensity of several CS isometric exercises. Additionally, this study analyzed to what extent the pelvic acceleration data represent the local stability of the core structures or the whole-body postural control. Twenty-three male and female physically-active individuals performed two testing-sessions spaced one week apart, each consisting of two 6-second trials of five variations of frontal bridge, back bridge, lateral bridge and bird-dog exercises. In order to assess load intensity based on the postural control challenge of CS exercises, a smartphone accelerometer and two force platforms were used to measure the mean pelvic linear acceleration and the mean velocity of the centre of pressure displacement, respectively. Reliability was assessed through the intra-class correlation coefficient (ICC_3,1_) and the standard error of measurement (SEM). In addition, Pearson coefficient was used to analyze the correlation between parameters. The reliability analysis showed that most CS exercise variations obtained moderate-to-high reliability scores for pelvic acceleration (0.71<ICC<0.88; 13.23%≤SEM≤22.99%) and low-to-moderate reliability scores for centre of pressure displacement (0.24<ICC<0.89; 9.88%≤SEM≤35.90%). Regarding the correlation analysis, correlations between pelvic acceleration and centre of pressure displacement were moderate-to-high (0.52≤*r*≤0.81). Based on these results, smartphone accelerometers seem reliable devices to quantify isometric CS exercise intensity, which is useful to identify the individuals’ CS status and to improve the dose-response characterization of CS programs.

## Introduction

Based on the results of correlational and experimental studies [1–4], core stability (CS) training is largely used in different fields nowadays, mainly to enhance athletic performance and to prevent and rehabilitate musculoskeletal injuries. However, in several experimental studies CS training programs have not delivered as positive results as could be expected [5–7]. One of the main reasons which could explain these poor and controversial results is the limited modulation and quantification of the training load parameters, especially the training intensity. In CS programs, training volume has been modulated through easily quantifiable parameters, i.e. exercise duration, number of repetitions and sets, etc. [5, 8, 9]. However, although training intensity has been manipulated by modifying the CS exercise difficulty through variations in different mechanical constraints (i.e. participant posture, lever arms, base of support, unstable surfaces, etc.) [9–11], to the best of the authors’ knowledge no experimental study has quantified the CS training intensity based on objective parameters.

The quantification of the load intensity is essential to analyze the dose-response relationships between training and CS adaptations. Coaches, fitness instructors, practitioners and researchers usually manipulate the CS exercise intensity based on their personal criteria but they do not use any field-based methodology or technique to assess whether the level of difficulty of the CS exercises is sufficient to challenge the stability of the core structures and thus, to induce CS adaptations [12]. In laboratory settings, the participants’ difficulty to maintain or resume a desired posture or trajectory of the trunk is accurately evaluated using biomechanical methods, such as sudden loading [13–16] and/or balancing protocols in seated positions [13, 14, 17–19]. However, these methods do not seem to be suitable to quantify CS training load, as they have a high-cost and complex data processing, and especially because their outcomes are not obtained during the execution of the CS exercises and therefore they are not easily applicable to training prescription. Among the different laboratory instruments, accelerometers might be able to overcome these drawbacks, as they have some features that make them a potential tool to assess CS while performing these exercises. Nowadays, accelerometers are integrated into electronic devices such as smartphones and iPods [20, 21], and this has turned them into suitable devices that can be used in professional and scientific applications because of their low cost, portability and ease of use. In addition, smartphone accelerometers have already proven their reliability quantifying stability in different balance conditions [22, 23]. However, to the authors’ knowledge there are no studies on the suitability of these accelerometers to quantify the CS training intensity based on the postural control challenge of the exercises.

In the current study, several of the most common CS exercises employed in fitness, sports and rehabilitation (frontal bridge, back bridge, lateral bridge and bird-dog) [24] were performed with a smartphone accelerometer placed on the pelvis while carrying out the exercises on two force platforms. Pelvis accelerations were used as measures of CS based on the lumbopelvic stability concept developed in clinical settings, in which CS has usually been evaluated as the ability to maintain a given lumbopelvic position in lying supine during different exercises [9, 25, 26]. The main objective was to evaluate the reliability of the smartphone accelerometer to quantify the intensity of these CS exercises. Additionally, the relationship between pelvic accelerations and whole-body postural control (i.e. center of pressure (COP) sway) was also analyzed to enable a discussion about local and global stability. Overall, the obtainment of an accurate and reliable tool to quantify the intensity of CS exercises would allow both to identify the individuals’ CS level and to manipulate training loads during CS interventions. This may be helpful for a dose-response characterization of CS training programs.

## Materials and methods

### Participants

Twenty-three healthy male (n = 12; age: 23.5±3.6 years; height: 173.9±4.7 cm; mass: 73.9±6.3 kg) and female (n = 11; age: 24.1±1.5 years; height: 165.0±11.5 cm; mass: 63.1±8.8 kg) volunteers participated in the study. In an attempt to minimize the potential variability caused by the individuals’ physical condition, all participants were physically-active with a work-out frequency of 2–3 days per week and their age ranged from 18 to 30 years. Additionally, due to the dimensions of the force platforms participants’ height was limited to a maximum of 185 cm, which also helped to reduce the influence of the anthropometry on the posturographic data. Pregnant females and participants with inguinal hernia, urinary incontinence or any pathology that contraindicated physical exercise practice were excluded from the study. Participants filled out a written informed consent in accordance with the Declaration of Helsinki. The study protocol was approved by the University Office for Research Ethics (DPS.FVG.02.14) at the Miguel Hernandez University of Elche (Spain).

### Experimental procedure

**Fig 1:**
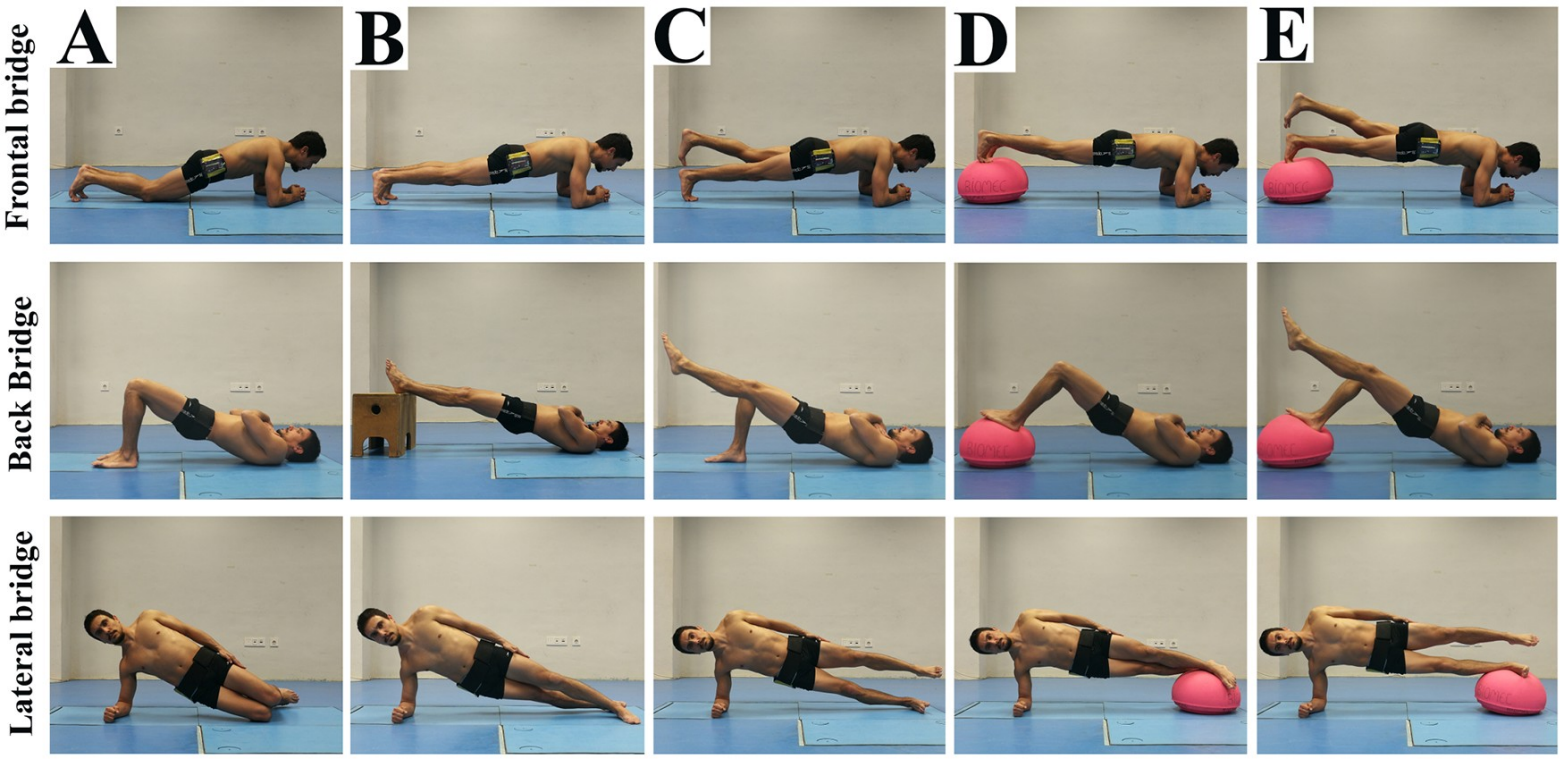
Variations of the frontal, dorsal and lateral bridge exercises. A) short bridges; B) long bridges; C) bridging with single leg support; D) bridging with double leg support on an unstable surface; E) bridging with single leg support on an unstable surface. The depicted individual is the first author. The individuals in this manuscript have given written informed consent (as outlined in PLOS consent form) to publish these case details.

**Fig 2:**
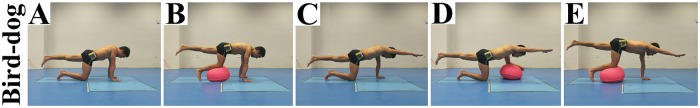
Variations of the bird-dog exercise. A) three-point position with an elevated leg; B) three-point position with an elevated leg and the contralateral knee on an unstable surface; C) classic two-point bird-dog position with elevated contralateral leg and arm; D) two-point bird-dog position with the forearm on an unstable surface; E) two-point bird-dog position with the knee on an unstable surface. The depicted individual is the first author. The individuals in this manuscript have given written informed consent (as outlined in PLOS consent form) to publish these case details.

The participants completed two testing sessions (60 min each) spaced one week apart in a biomechanics laboratory. In each testing session, the participants performed five variations of the four CS exercises twice (Figs 1 and 2), for a total of 40 trials. For the bridging exercises, the following variations were performed based on a progression established through changes in the gravitational torque on the trunk, the number of supporting limbs and/or the use of an unstable surface (i.e. BOSU^TM^ balance trainer) (Fig 1): A) short bridges, B) long bridges, C) bridging with single leg support, D) bridging with double leg support on an unstable surface, and E) bridging with single leg support on an unstable surface. As the bird-dog has different characteristics, the following progression was performed (Fig 2): A) three-point position with an elevated leg, B) three-point position with an elevated leg and the contralateral knee on an unstable surface, C) classic two-point bird-dog position with elevated contralateral leg and arm, D) two-point bird-dog position with the forearm on an unstable surface, and E) two-point bird-dog position with the knee on an unstable surface. All the variations performed on a single leg were carried out with dominant limb support.

To analyze whole-body postural control during the CS exercises, each trial was carried out on two synchronized force platforms (9287CA, Kistler, Switzerland). The COP displacement was recorded at 1000 samples/s in anterior-posterior and medial-lateral directions through the BioWare software (version 5.2.1.3, Kistler, Switzerland). At the same time, to assess lower-trunk postural control, pelvic linear accelerations were recorded at 100 samples/s from a 3-axis accelerometer (model LIS3DH, STMicroelectronics, Switzerland) embedded in a smartphone (Motorola Moto G, 2013, USA), using a free mobile application (Accelerometer Analyzer, Mobile Tools, Poland) from which earth gravity was removed. An adjustable belt was used to place the smartphone on the dominant side of the pelvis, between the iliac crest and the great trochanter. This location was chosen to reduce accelerometer motions caused by muscle contractions. The accelerometer onset was remotely controlled from a computer through a free application (TeamViewer QuickSupport, TeamViewer, Germany). This computer was also used to collect the COP data simultaneously.

Prior to testing, participants performed a warm-up, which consisted of 10 repetitions of the following exercises: lumbo-pelvic mobility (i.e. pelvic circles, pelvic anteversion and retroversion, and cat-camel), twisting crunch, side crunch, trunk extension and free-weight squat. During the testing trials, frontal bridge, back bridge, lateral bridge and bird-dog variations were performed under the instruction that trunk motion was to be maintained to a minimum, while keeping the lumbar spine and pelvis in a “neutral” position. In each trial, a researcher placed the participants in the proper position, which they had to hold for 6 s, with a 60-second rest between trials. This short exercise duration was chosen to reduce the influence of muscle fatigue on postural control throughout the 40 trials performed in each testing session. The order of the four exercise progressions (frontal bridges, back bridges, lateral bridges and bird-dogs) was randomized between participants. Additionally, in each progression half of the sample performed the five exercise variations from the easiest to the most difficult condition and vice versa.

### Data processing

The first and last second of each trial were discarded, analyzing a 4 s window for both COP and acceleration time series. COP data of both force platforms were unified through the algorithm proposed by the product supplier and low-pass filtered at 5 Hz (4^th^-order, zero-phase-lag, Butterworth) [27]. Then, the mean velocity of COP displacement was computed [28]. Regarding the pelvic linear acceleration, the smartphone accelerometer signal was low-pass filtered at 10 Hz (4^th^-order, zero-phase-lag, Butterworth) [29] and the mean acceleration was calculated as the average of the acceleration magnitude data series [30, 31]. The computation of the COP and acceleration variables was carried out with “ad hoc” software, developed by our research group within LabView 9.0 environment (National Instruments, USA).

### Statistical analysis

The descriptive data of each variable were presented as mean and standard deviations. The normal distribution of the data was confirmed using the Kolmogorov–Smirnov test with the Lilliefors correction.

To analyze the relative and absolute reliability, the intra-class correlation coefficient (ICC_3,1_) and the standard error of measurement (SEM) were calculated, respectively [32]. ICC_3,1_ values were interpreted according to the following criteria: excellent (0.90–1.00), good (0.70–0.89), fair (0.50–0.69), low (<0.50) [33]. The SEM was calculated as the standard deviation of the difference between the two sessions divided by √2 [34]. This method was employed to reduce the impact of sample heterogeneity and the influence of systematic error. SEM was expressed as absolute values and percentages to facilitate data extrapolation. Taking into account that the magnitude of SEM variability is task-dependent [34], qualitative interpretation of SEM scores was based on reliability findings from previous posturographic studies. Typically, COP parameters display absolute reliability scores ranging from 10% to 30% [35, 36]. However, a reliability study on posturographic parameters [35] observed that only when SEM scores were lower than 20%, ICC scores were higher than 0.75. Thus, SEM values lower than 20% were considered acceptable for this study. The interval confidence limits were calculated at 95% for ICC and SEM. Reliability analyses were carried out using a spreadsheet designed by Hopkins [37]. Because the storage memory of the smarphone was not freed up during the first recording session, pelvis acceleration data from 6 participants were missed. Therefore, the reliability analysis of the smartphone acceleration data was carried out with 17 participants.

To assess the possible existence of learning effect in the measurements, a one-way repeated measures ANOVA being the *measurement* the within-subject factor (2 levels: test and retest) were employed to compare the COP and acceleration variables between testing sessions. The practical significance of the learning effect was assessed by calculating Cohen’s effect size with Hedges’ adjustment [38]. Effect sizes >0.8, 0.8–0.5, 0.5–0.2 and <0.2 were considered large, moderate, small, and trivial, respectively [39].

In addition, the possible relationships between the COP and acceleration variables were evaluated using the Pearson correlation coefficient. The SPSS package (version 22, SPSS Chicago, Illinois, USA) was used to perform the ANOVA and correlational analysis, with the significance level set at 0.05.

Before ANOVA and reliability analysis, a power analysis was performed to calculate the minimum sample size needed to detect significant results. For the reliability analysis, based on Walter et al.’s algorithm [40], a sample size of 22 participants (17 excluding dropouts) was necessary to find ICC scores ≥ 0.7 as significant results (*alternative hypothesis*: ICC≥0.7; *null hypothesis*: ICC≤0.4; number of observations per subject = 4; power = 80%; α = 0.05; possible dropout = 20%). For the ANOVA, the free sampling software package GPower 3.1. [41] was used to estimate the minimum sample size, showing that a sample size of 12 participants (10 excluding dropouts) was needed to detect subtle within-group significant differences (effect size = 0.3) caused by learning in the test-retest assessment (r = 0.6; power = 80%; α = 0.05; possible dropout = 20%).

## Results

Overall, the absolute and relative reliability shown by the mean velocity of COP displacement ranged from low to moderate for most CS exercise variations (Table 1). In this sense, only eight out of 20 exercise variations displayed an adequate reliability (ICC≥0.70; SEM≤20%). On the other hand, as shown in Table 2, the mean pelvic acceleration presented good relative reliability for most exercise variations (0.71<ICC<0.88), except for the three-point bird-dog position with an elevated leg which only obtained fair values (ICC = 0.62). In addition, the mean pelvic acceleration showed adequate absolute reliability scores in 14 out of 20 CS exercise variations (13.23%≤SEM≤19.97%), obtaining an average for all exercise variations of 0.080 m/s^2^ (confidence limits at 95%: 0.066–0.093 m/s^2^). Lateral-bridge variations showed the best SEM scores, without any variation showing SEM scores above 20%. Conversely, three of the five bird-dog variations showed SEM values higher than 20%. Concerning the learning effect analysis, mean velocity of COP displacement and mean pelvic acceleration showed no significant differences (p>0.05) between days for most CS exercise variations.

**Table 1:** Descriptive statistics (mean ± SD) and relative (ICC_3,1_) and absolute (SEM) between-session reliability for the mean velocity of center of pressure displacement (mm/s) obtained during the different variations of the trunk stabilization exercises.

Exercises	Variations	Session 1	Session 2	t	*p*	*d*	SEM (mm/s)			ICC_3,1_	
							Mean	(LCL–UCL)	%	Mean	(LCL–UCL)
Back Bridge*	**A**	17.18 ± 5.71	17.36 ± 4.16	-0.14	0.89	0.04	4.39	3.38–6.27	25.42	0.24	-0.19–0.59
	**B**	27.30 ± 8.43	27.06 ± 8.72	0.12	0.91	-0.03	7.22	5.55–10.32	26.56	0.31	-0.12–0.64
	**C**	32.09 ± 9.95	32.22 ± 8.17	-0.07	0.95	0.01	6.14	4.75–8.69	19.09	0.56	0.21–0.79
	**D**	36.32 ± 10.71	33.58 ± 11.23	1.68	0.11	-0.25	5.54	4.29–7.84	15.86	0.76	0.52–0.89
	**E**	46.38 ± 15.18	46.31 ± 14.49	0.05	0.97	-0.01	5.70	4.41–8.07	12.30	0.86	0.71–0.94
Frontal Bridge*	**A**	15.80 ± 7.46	15.50 ± 6.11	0.18	0.86	-0.04	5.62	4.32–8.03	35.90	0.34	-0.09–0.66
	**B**	26.88 ± 8.59	28.99 ± 10.25	-1.35	0.19	0.22	5.31	4.11–7.52	19.02	0.70	0.42–0.86
	**C**	40.73 ± 13.46	39.30 ± 13.71	0.54	0.60	-0.11	9.02	6.98–12.77	22.54	0.58	0.23–0.80
	**D**	47.72 ± 16.32	44.84 ± 14.44	1.25	0.22	-0.19	7.78	6.02–11.02	16.82	0.76	0.52–0.89
	**E**	50.54 ± 14.16	48.70 ± 14.24	0.68	0.50	-0.13	9.16	7.08–12.96	18.46	0.60	0.26–0.81
Lateral Bridge*	**A**	27.56 ± 8.18	24.05 ± 8.41	2.04	0.53	-0.42	5.82	4.50–8.23	22.54	0.53	0.16–0.77
	**B**	39.73 ± 9.21	40.00 ± 11.15	-0.14	0.89	0.03	6.49	5.02–9.18	16.28	0.62	0.28–0.82
	**C**	62.15 ± 18.82	59.35 ± 23.00	0.80	0.43	-0.13	11.81	9.13–16.71	19.44	0.70	0.42–0.86
	**D**	73.93 ± 25.09	65.63 ± 21.35	2.73	0.01	-0.36	10.31	7.97–14.59	14.78	0.82	0.62–0.92
	**E**	81.36 ± 22.96	71.74 ± 21.49	4.22	0.00	-0.43	7.56	5.82–10.81	9.88	0.89	0.76–0.95
Bird-Dog**	**A**	20.38 ± 6.87	19.39 ± 5.85	0.76	0.46	-0.16	4.45	3.44–6.30	22.39	0.53	0.16–0.77
	**B**	31.37 ± 9.22	29.22 ± 10.92	0.93	0.36	-0.21	7.84	5.42–9.93	25.89	0.41	0.42–0.86
	**C**	42.14 ± 11.99	40.19 ± 12.96	0.94	0.36	-0.16	7.01	8.10–15.04	17.04	0.70	-0.11–0.64
	**D**	54.36 ± 12.55	48.66 ± 12.61	1.80	0.87	-0.45	10.52	6.07–11.10	20.43	0.31	0.01–0.70
	**E**	55.50 ± 16.38	56.60 ± 14.57	-0.34	0.74	0.07	10.73	8.25–15.33	19.14	0.54	0.16–0.78

SD: standard deviation; SEM: standard error of measurement; %: SEM mean expressed in percentage; ICC_3,1_: intraclass correlation coefficient; LCL: lower confidence limit at 95%; UCL: upper confidence limit at 95%; *d*: effect size.

*Variations of the frontal, *dorsal and lateral bridge exercises*: A: short bridges; B: long bridges; C: bridging with single leg support; D: bridging with double leg support on an unstable surface; E: bridging with single leg support on an unstable surface.

**Variations of the bird-dog exercise: A: three-point position with an elevated leg; B: three-point position with an elevated leg and the contralateral knee on an unstable surface; C: classic two-point bird-dog position with elevated contralateral leg and arm; D: two-point bird-dog position with the forearm on an unstable surface; E: two-point bird-dog position with the knee on an unstable surface.

**Table 2:** Descriptive statistics (mean ± SD) and relative (ICC_3,1_) and absolute (SEM) between-session reliability for the mean acceleration (m/s^2^) of smartphone accelerometer obtained during the different variations of the trunk stabilization exercises.

Exercise	Variations	Session 1	Session 2	t	*p*	*d*	SEM (m/s^2^)			ICC_3,1_	
							Mean	(LCL–UCL)	%	Mean	(LCL–UCL)
Back Bridge*	**A**	0.25 ± 0.11	0.25 ± 0.09	0.03	0.98	0.00	0.05	0.04–0.08	20.93	0.76	0.45–0.91
	**B**	0.22 ± 0.07	0.23 ± 0.09	-0.32	0.76	0.05	0.04	0.03–0.06	18.57	0.77	0.48–0.91
	**C**	0.60 ± 0.21	0.54 ± 0.16	2.29	0.04	-0.33	0.08	0.06–0.12	13.23	0.84	0.62–0.94
	**D**	0.43 ± 0.20	0.39 ± 0.17	1.01	0.33	-0.18	0.10	0.07–0.15	22.50	0.76	0.45–0.91
	**E**	0.57 ± 0.23	0.57 ± 0.21	0.01	0.99	0.00	0.08	0.06–0.12	14.42	0.88	0.70–0.95
Frontal Bridge*	**A**	0.17 ± 0.05	0.18 ± 0.05	-0.49	0.63	0.07	0.02	0.02–0.03	12.21	0.85	0.63–0.94
	**B**	0.31 ± 0.14	0.35 ± 0.18	-1.33	0.20	0.21	0.07	0.05–0.11	22.99	0.82	0.56–0.93
	**C**	0.57 ± 0.25	0.53 ± 0.24	1.18	0.26	-0.18	0.11	0.08–0.16	18.63	0.83	0.59–0.93
	**D**	0.39 ± 0.17	0.38 ± 0.14	0.50	0.63	-0.07	0.06	0.05–0.09	15.94	0.86	0.66–0.95
	**E**	0.65 ± 0.27	0.61 ± 0.23	1.38	0.19	-0.17	0.09	0.07–0.14	14.22	0.88	0.70–0.95
Lateral Bridge*	**A**	0.29 ± 0.09	0.27 ± 0.08	1.39	0.18	-0.24	0.04	0.03–0.06	14.50	0.77	0.48–0.91
	**B**	0.51 ± 0.20	0.48 ± 0.17	0.97	0.35	-0.17	0.09	0.07–0.14	18.60	0.77	0.46–0.91
	**C**	0.57 ± 0.21	0.58 ± 0.22	-0.14	0.89	0.02	0.11	0.08–0.17	19.39	0.75	0.44–0.90
	**D**	0.58 ± 0.20	0.59 ± 0.22	-0.26	0.80	0.04	0.10	0.08–0.16	17.95	0.78	0.49–0.91
	**E**	0.75 ± 0.29	0.66 ± 0.20	1.97	0.07	-0.36	0.13	0.10–0.20	17.61	0.74	0.41–0.90
Bird-Dog**	**A**	0.26 ± 0.11	0.26 ± 0.09	0.37	0.72	-0.08	0.07	0.05–0.10	25.36	0.62	0.21–0.84
	**B**	0.35 ± 0.12	0.34 ± 0.14	0.22	0.83	-0.04	0.07	0.05–0.10	21.32	0.71	0.40–0.89
	**C**	0.33 ± 0.12	0.34 ± 0.12	-0.45	0.66	0.08	0.07	0.07–0.14	19.97	0.73	0.41–0.90
	**D**	0.52 ± 0.20	0.47 ± 0.14	1.81	0.09	-0.33	0.09	0.06–0.11	17.97	0.74	0.36–0.88
	**E**	0.57 ± 0.21	0.57 ± 0.21	0.10	0.92	-0.02	0.12	0.09–0.18	20.85	0.71	0.36–0.88

SD: standard deviation; SEM: standard error of measurement; %: SEM mean expressed in percentage; ICC_3,1_: intraclass correlation coefficient; LCL: lower confidence limit at 95%; UCL: upper confidence limit at 95%; *d*: effect size.

*Variations of the frontal, *dorsal and lateral bridge exercises*: A: short bridges; B: long bridges; C: bridging with single leg support; D: bridging with double leg support on an unstable surface; E: bridging with single leg support on an unstable surface.

**Variations of the bird-dog exercise: A: three-point position with an elevated leg; B: three-point position with an elevated leg and the contralateral knee on an unstable surface; C: classic two-point bird-dog position with elevated contralateral leg and arm; D: two-point bird-dog position with the forearm on an unstable surface; E: two-point bird-dog position with the knee on an unstable surface.

Finally, moderate to high correlations (0.52≤*r*≤0.81) were found between mean velocity of COP displacement and mean pelvic acceleration during the CS exercise variations(Table 3).

**Table 3:** Pearson correlation moment between mean acceleration of smartphone accelerometer (m/s^2^) and mean velocity of center of pressure displacement (mm/s) obtained during the different variations of the trunk stabilization exercises.

*Variations	Back Bridge	Frontal Bridge	Lateral Bridge	Bird-Dog
**A**	0.58	0.56	0.79	0.85
**B**	0.76	0.83	0.64	0.80
**C**	0.47	0.76	0.67	0.82
**D**	0.60	0.69	0.83	0.75
**E**	0.77	0.84	0.78	0.67
**Mean ± SD**	0.63 ± 0.13	0.74 ± 0.12	0.74 ± 0.08	0.78 ± 0.06

*Variations for the bridge exercises: A: short bridge; B: long bridge; C: bridging with single leg support; D: bridging with double leg support on an unstable surface; E: bridging with single leg support on an unstable surface. *Variations for the bird-dog exercise*: A: three-point position with an elevated leg; B: three-point position with an elevated leg and the contralateral knee on an unstable surface; C: classic two-point bird-dog position with elevated contralateral leg and arm; D: two-point bird-dog position with the forearm on an unstable surface; E: two-point bird-dog position with the knee on an unstable surface.

SD: standard deviation.

## Discussion

One of the main limitations of CS training programs is the lack of methods to quantify the intensity of the CS exercises, which hinders the design of these programs and the comparison and generalization of their effects. The aim of this study was to examine the relative and absolute reliability of accelerometers embedded in smartphones for the quantification of CS training intensity based on the postural control challenge of the exercises. Additionally, acceleration data were correlated to COP parameters to analyze to what extent smartphone accelerometer measures reflect the local stability of the core structures or the whole-body postural control.

The main results of our study showed that smartphone accelerometers are reliable tools to quantify the postural control challenge of the CS exercises, displaying high reliability scores in most exercises (ICC≥0.70; SEM≤20%) and supporting the use of the accelerometers in balance studies [21, 42–44]. These results together with the low-cost and portability of smartphones could lead the design of CS training programs to a more quantitative approach. In this sense, the high relative reliability displayed by the acceleration data shows the smartphone consistency to objectively rank individuals [32], which would facilitate the individualization of intervention programs according to each person’s CS status. Additionally, absolute reliability scores provided reference cut-offs to discriminate if longitudinal changes in pelvic sway during CS exercises are caused by within-subject day-to-day variability or by real changes in CS status [34]. Specifically, based on the average of SEM scores and its confidence limits at 95%, reductions higher than 0.1 m/s^2^ would reflect a real improvement caused by CS interventions in most exercises.

Although force platforms have been successfully applied for postural control evaluation in different conditions [28, 36], mean velocity of the COP in this study mostly displayed moderate to low reliability results (Table 1). Interestingly, some of the most challenging exercise variations (e.g. lateral bridge with single leg support on an unstable surface) displayed the best reliability scores, probably because the increase of neuromuscular control demands reduced outcome variability [13, 45]. Probably, the low reliability of many of the COP variables was caused by the short duration of the trials performed in the current study (6 s), leading to the non-stationary behavior of COP displacements, which could cause the capture of only part of the individuals’ dynamic oscillations [45], consequently resulting in high within-subject variability [36, 46]. Considering the good reliability displayed by the smartphone accelerometer, acceleration data seemed to be less influenced by the non-stationarity of postural control in the short-term [45], which allows to obtain a reliable short time assessment of CS without the influence of muscle fatigue on postural control. Moreover, this short exercise duration reduced the data collection period, which additionally helped to minimize the learning effect of the exercises, as was confirmed by the low differences in the amplitude of pelvic accelerations between testing session 1 and 2 (Table 2).

The results of the correlational analysis reinforce the use of smartphone accelerometers for quantifying CS (Table 3). Although the correlations between COP and acceleration parameters were moderate to high (0.52≤*r*≤0.81), the explained variance between variables only ranged from 27.0% to 65.6% and therefore both parameters probably do not measure the same postural control capability [20]. Thus, taking into account that COP displacement during static balance tasks is associated to the neuromuscular responses derived from the body’s center of mass motion [47], COP parameters would reflect the individuals’ whole-body postural control. Conversely, as the smartphone accelerometer was placed on the pelvis, acceleration data (i.e. pelvic sway) would be more related to the local postural control [20] of the core structures and consequently it would be more useful to quantify the intensity of CS exercises.

**Fig 3:**
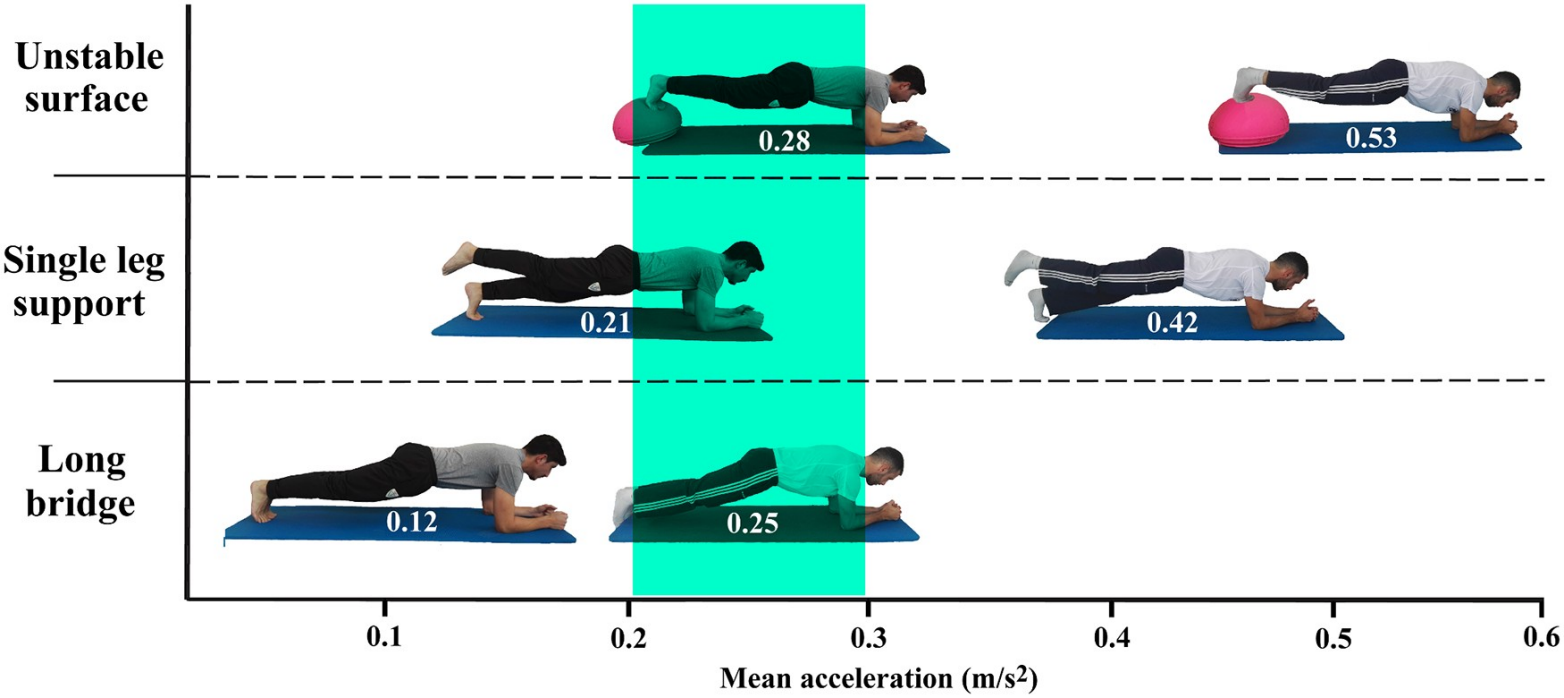
Pelvic mean acceleration values obtained with a smartphone accelerometer in two participants during the execution of three variations of the frontal bridge. The individuals in this picture have given written informed consent (as outlined in PLOS consent form) to publish these case details.

One of the most interesting applications of the results of this study is that smartphone accelerometers allow an objective and reliable assessment of the participants’ performance during some of the most popular CS exercises, which may facilitate training intensity quantification during CS programs. For example, as shown in Fig 3, the acceleration values provided by the smartphone may help to individually quantify the intensity of several variations of the frontal bridge according to the magnitude of pelvic accelerations, which reflect the postural control challenge imposed on each participant. This information could be used to establish CS exercise progressions and to choose those exercises that produce the desired intensity level for each participant. Interestingly, as Fig 3 and S1 Video show, similar intensity levels (e.g. 0.2–0.3 m/s^2^) can be achieved using different exercises depending on the participant’s characteristics. However, in most CS training programs found in the literature all participants performed the same exercises [5, 48, 49], while the exercise intensity was not quantified; consequently, many participants could have trained at different intensity levels (S2 Video), eliciting different neural and/or physiological responses and inducing different adaptations [12]. In order to obtain a proper dose-response characterization of CS training programs, future studies could use smartphone accelerometers to explore the effects of different training intensities and progressions in several populations. Possibly, the use of high intensity CS exercises (i.e. exercises that mainly challenge the participants’ postural control) would produce higher stability adaptations than longer CS exercises performed at low-moderate intensity levels (i.e. exercises that mainly challenge the participants’ endurance). However, further research is needed to test this hypothesis and to determine which acceleration levels are the most suitable to increase CS in each population.

To our knowledge, this is the first study using a smartphone accelerometer to quantify CS training intensity based on the postural control challenge of the exercises. Nonetheless, the current results must be interpreted with caution as this study has some limitations. For instance, generalization of the data in our study is limited because our participants were young and physically active. In this sense, even though the accelerometer showed good reliability to measure pelvic sway during different CS exercises, future studies should analyze the consistency of this device in other populations and CS exercises. In addition, even though accelerometers offer an objective CS assessment, they do not provide information about the spine position, so it is possible that in some trials participants did not maintain the spine in neutral position. It should be noted that smartphone accelerometers can help, but not replace trainers’ labor, as during their use in CS exercises it necessary to check the individuals’ exercise technique.

## Conclusions

Smartphone accelerometers are reliable tools to quantify CS training intensity based on the postural control challenge of the isometric CS exercises. Considering their relative and absolute reliability scores, low-cost, portability and usability, they seem suitable devices to objectively individualize intervention programs according to the participants’ CS status and to monitor the effectiveness of CS training programs in research and clinical settings. In addition, taking into account the correlation analysis, smartphone accelerometers placed on the pelvis provide local measures of postural control of the core structures rather than global measures of whole-body postural control.

## Supporting information

S1 Video Different core stability isometric exercises elicit similar pelvis acceleration in two individuals. The individuals in this manuscript have given written informed consent (as outlined in PLOS consent form) to publish these case details.
(MP4)

S2 Video The same core stability isometric exercise elicits different pelvis acceleration in two individuals. The individuals in this manuscript have given written informed consent (as outlined in PLOS consent form) to publish these case details.
(MP4)

S1 File Study database. (XLSX)
